# Development of a hub-and-spoke durable left ventricular assist device program in Brazil, a middle-income country

**DOI:** 10.1016/j.jhlto.2024.100151

**Published:** 2024-08-23

**Authors:** Deborah de Sá Pereira Belfort, Bruno Biselli, Mônica Samuel Avila, Renata Lopes Hames, Stephanie Itala Rizk, Fabrício Canova Calil, Bruna Carneiro Oliveira, Filomena Regina Barbosa Gomes Galas, Ludhmila Abrahão Hajjar, Nadine Oliveira Clausell, Livia Adams Goldraich, Ramez Anbar, Edimar Alcides Bocchi, Tadeu Thomé, Roberto Kalil Filho, Paulo Manuel Pêgo-Fernandes, Fabio Biscegli Jatene, Silvia Moreira Ayub-Ferreira

**Affiliations:** a Hospital Sirio-Libanes, São Paulo, SP, Brazil; b Heart Institute (InCor), Instituto do Coração, Hospital das Clinicas, HCFMUSP, Universidade de São Paulo, São Paulo, SP, Brazil; c Hospital de Clinicas de Porto Alegre, Universidade Federal do Rio Grande do Sul, Porto Alegre, RS, Brazil

**Keywords:** left ventricular assist device, survival analysis, middle-income countries, hub-and-spoke, heart failure

## Abstract

**Background:**

In middle-income countries, costs limit widespred use of left ventricular assist device (LVAD) as a strategy for end-stage heart failure. We aim to describe the experience of a LVAD program in a middle-income country in a hub-and-spoke model.

**Methods:**

Patients fulfilling strict inclusion and exclusion criteria were referred from different centers in Brazil for LVAD implantation through a philanthropy program financed via a Brazilian Federal Government tax exemption structure. LVAD implantation was performed in a hub-and-spoke model with a single implanting center. Data were collected retrospectively using hospital records and telephone contact with other centers. Patients who received LVAD implants external to the philanthropic program, either at this or other Brazilian centers, were not included.

**Results:**

Between January 1, 2013 and December 31, 2020, 20 adult patients underwent long-term continuous flow LVAD implantation with decentralization of postimplant patient care in regional centers. Patients were referred from 11 centers from 7 states in Brazil and underwent LVAD implantation through a philanthropy program. The median age was 52.5 years and 85% were Interagency Registry for Mechanically Assisted Circulatory Support profile 3 patients. Two patients had Chagas cardiomyopathy. The overall survival censored for competing risks at 1 and 2 years were 90% and 84%, respectively. Three patients (15%) underwent heart transplantation in the first 2 years after LVAD implantation. Twelve patients returned to their original centers and were followed remotely.

**Conclusions:**

This study presents a successful LVAD implantation program in a hub-and-spoke model in Brazil. Centralization of LVAD implantation with decentralization of postimplant patient care in regional centers is feasible and safe, enabling optimal allocation of resources in middle-income countries.

## Background

The majority of left ventricular assist devices (LVADs) are currently implanted in high-income countries.^1^, ^2^ In middle-income countries, costs limit their widespread use as a strategy for patients with end-stage heart failure (HF). Brazil is a country with a mature heart transplantation (HT) network, with most HTs being performed through the Brazilian public health system—the Brazilian Universal Healthcare System (Sistema Único de Saúde [SUS]), but the annual number of HTs is still below the needs of the country.^3^ Whereas SUS provides universal coverage for HT, SUS does not provide for coverage of LVAD implantation; instead LVAD therapy may be accessed selectively and with effort by commercially-insured or wealthy individuals in a select number of private hospitals in certain regions of Brazil.

To expand LVAD access in our country, a philanthropy program for LVAD implantation was developed in partnership with Hospital Sírio Libanês, a philanthropic hospital located in São Paulo, Brazil. This program is part of the Support Program for Institutional Development of the Unified Health System (Programa de Apoio ao Desenvolvimento Institucional do SUS), a program conducted in partnership with philanthropic hospitals of recognized quality financed from tax expenditures, which are indirect expenditures from the Federal Government’s tax exemption.^4^ In this program, end-stage HF patients are referred from regional care centers in Brazil to have an LVAD implanted at Hospital Sírio Libanês, allowing patients over greater geographical distances to receive access to this treatment.

In this article, we describe the experience of LVAD implantation in a hub-and-spoke model in Brazil through a philanthropy program over a period of 8 years.

## Materials and methods

### Inclusion and exclusion criteria

Patients implanted with a long-term continuous flow LVAD between January 1, 2013 and December 31, 2020 were included in this retrospective analysis. Patients implanted via this philanthropy program were carefully evaluated by a multidisciplinary LVAD team of HF specialists, cardiac surgeons, and allied professionals, including LVAD coordinators, social workers, psychologists, and nutritionists. Adult patients between the ages of 18 and 65 years (inclusive) fulfilling physiologic criteria for Interagency Registry for Mechanically Assisted Circulatory Support (INTERMACS) profile 3 and requiring inotropes for at least 14 days were selected and implanted with long-term LVAD support with bridge-to-transplant (BTT), bridge-to-candidacy (BTC), and destination therapy (DT) intent. This included BTT patients listed for HT with an anticipated long waiting time (for example, patients highly immunologically sensitized), BTC patients with potentially modifiable current contraindications for HT (such as pulmonary hypertension, obesity, or history of cancer treatment without yet achieving cure criteria), and DT patients with enduring or definitive contraindications to HT. In exceptional circumstances, INTERMACS profile 2 patients were also accepted for LVAD implant. Although there are a few literature reports of LVAD implants Chagas cardiomyopathy, patients with dilated cardiomyopathy due to Chagasic cardiomyopathy were also included. While left ventricular systolic function (LVEF) of ≤25% is typically required, we did not use this cutoff as all patients were receiving inotropic agents, and this may acutely augment LVEF. Outpatient continuous infusion of systemic inotropes is generally unavailable in Brazil, rendering such patients hospital-confined through implant.

The exclusion criteria were acute or chronic kidney failure with severely decreased kidney function or kidney failure (creatinine >3.0 mg/dl, creatinine clearance <30 ml/min/1.73 m^2^ or hemodialysis); hepatic dysfunction (bilirubin >2.5 mg/dl, alanine aminotransferase or aspartate aminotransferase >3× normal range or International Normalized Ratio [INR] >2.5 in the absence of anticoagulation); body mass index <21 kg/m^2^ for men and <19 kg/m^2^ for women; recent stroke; severe neuropsychiatric deficit that precludes LVAD management; severe vascular peripheral disease; active systemic infection; severe pulmonary disease (forced expiratory volume in the first second <1 liter); multiple organ failure; pulmonary infarction in the last 6 weeks; contraindication for anticoagulation; heparin-induced thrombocytopenia; class 3 obesity (body mass index >40 kg/m^2^); invasive mechanical ventilation, including extracorporeal membrane oxygenation requirement; severe mitral stenosis or severe aortic insufficiency; and high surgical risk, including INTERMACS profile 1 patients. Left ventricular end-diastolic diameter was not an exclusion criterion.

### Anticoagulation and antiplatelet protocol

Patients who received a Berlin Heart INCOR (Berlin Heart GmbH, Berlin, Germany), an axial flow pump, were prescribed dual antiplatelet therapy following device’s protocol (acetylsalicylic acid [ASA] 100 mg once daily and dipyridamole 150 mg once daily) plus warfarin targeting INR between 2 and 3. Patients who received a HeartMate II (HMII—Abbott Laboratories, Chicago, IL) or HeartMate 3 (HM3—Abbott Laboratories, Chicago, IL) received single antiplatelet therapy with ASA 100 mg once daily plus warfarin to target INR 2 to 3 for HMII and ASA 100 mg once daily plus warfarin to target 2.0 to 2.5 for HM3. In cases where we deemed a higher risk of bleeding, we aimed for a target INR of 1.8 to 2.5.

### Patient referral and follow-up protocol

Patients enrolled in this program were covered by the public health care system and were referred from hospitals located in various cities across Brazil. The main reasons for referral were either the unavailability of LVADs at the regional center or the availability of LVAD implantation at centers solely for private patients. Local teams were responsible for selecting possible LVAD candidates, and each selected patient was discussed with the central center HF team to evaluate if patients fulfilled inclusion and exclusion criteria. If so, the patient was referred from local center to have LVAD implantation performed in the central center in a hub-and-spoke model.

After implantation, stabilization, and rehabilitation, patients were contra-referred to the regional centers. There were 2 different strategies of follow-up:

• If the local center was equipped with HF specialists, the patient was followed only in the regional center since these centers were equipped with specialized nurses, backup controllers, and the necessary structure for clinical interventions regarding LVAD recipient’s care. The central center served as a backup in case surgical intervention (e.g., LVAD exchange due to malfunction) was required.
• If the local center lacked HF specialists, patients were required to remain in São Paulo (where the central center was located) for at least 2 months after discharge. During this time, they received weekly follow-up care at the specialized LVAD outpatient clinic of the central center. Upon completion of this period, assuming the patient remained stable, without unresolved complications, and adherent to optimized HF medications and proper anticoagulation, they were permitted to return to their city of residence. Outpatient visits were then scheduled every 4 months at the central center, or more frequently if needed. Each local center had a designated cardiologist trained to oversee the ongoing care of these patients. Additionally, the central center provided monthly telemonitoring and offered 24-hour assistance from an LVAD coordinator to the local center.

### Data collection and statistical analysis

Preimplant patient characteristics such as sex, age, etiology of cardiomyopathy, strategy for implantation, and clinical status were recorded. The possible strategies for implantation were DT, BTC, and BTT. We divided the group BTT into 2 subgroups: BTT-unlikely, in which we allocated patients with total human leucocyte antigen class I and II calculated panel reactive antibody >80%, and BTT-likely, with the other patients. Patients were considered for the BTC strategy if they had contraindications to HT due to pulmonary hypertension unresponsive to pharmacological intervention or a history of cancer treatment with a disease-free period of less than 5 years.

Perioperative data, adverse events, and outcomes are also reported until December 31, 2022. For all adverse events, we utilized the updated International Society for Heart and Lung Transplantation definition of adverse events.^5^ Complications were considered early when they occurred within 30 days following the implant, while late complications were defined as those that occurred 30 days or later. Data were collected retrospectively using hospital records of our center and by contacting HF specialists at the different centers by telephone. No patients were lost to follow-up.

For descriptive purposes, continuous variables are expressed as medians and interquartile ranges, and categorical variables are expressed as frequencies and percentages. Kaplan-Meier survival estimates were calculated, and patients were censored at the time of transplantation or explant or inactivation of LVAD due to recovery. Median survival was expressed in days. Patients who had the device exchanged for another LVAD were not censored. For survival analyses, specific subsets were compared using log-rank testing.

The project was approved by the local Ethics Committee on July 13, 2020 (#1799; CAAE 33484320.5.0000.5461).

## Results

Between January 1, 2013 and December 31, 2020, a total of 20 adult patients underwent long-term continuous flow LVAD implantation in this philanthropy program.

### Patient characteristics

Baseline clinical data are presented in Table 1. The median age was 52.5 years, 12 (60%) patients were female and ischemic cardiomyopathy was the most common etiology for HF. All patients were on inotropes and 7 (35%) patients were supported by an intra-aortic balloon pump before implant. Estimated creatinine clearance was below 60 ml/min/1.73 m^2^ in 10 (50%) patients. BTT and BTC were the only strategies utilized in practice; no patient had an LVAD implanted with a definitive DT intent. Echocardiographic and hemodynamic data recorded 1 day before implantation are described in Table 2. All patients were on inotropes at the time echocardiogram and right heart catheterization were performed. The median LVEF was 25% (interquartile range 21%-28%, minimum LVEF 13% and maximum 31%). Only 1 patient had severe right ventricular dysfunction on echocardiogram; however, hemodynamic data suggested adequate right ventricular function, and this patient was included in the program after HF team evaluation.

**Table 1:** Baseline Characteristics

Baseline Characteristics	Total (N = 20)
Male *n* (%)	8 (40%)
Age (years)	52.5 (42.2-56)
Etiology of heart failure	
Ischemic *n* (%)	7 (35%)
Chagas *n* (%)	2 (10%)
Dilated *n* (%)	6 (30%)
Hypertensive *n* (%)	1 (5%)
Cardiotoxicity *n* (%)	3 (15%)
Other *n* (%)	1 (5%)
Comorbidities	
Hypertension *n* (%)	8 (40%)
Diabetes mellitus *n* (%)	3 (15%)
CAD *n* (%)	7 (35%)
Atrial fibrillation *n* (%)	6 (30%)
Previous sternotomy *n* (%)	0 (0%)
Previous stroke *n* (%)	2 (10%)
ICD/CRT *n* (%)	5 (25%)
Estimated glomerular filtration rate <60 ml/min/1.73 m^2^*n* (%)	10 (50%)
Hemodynamic support	
Dobutamine *n* (%)	18 (90%)
Milrinone *n* (%)	13 (65%)
Nitroprusside *n* (%)	4 (20%)
Vasopressor *n* (%)	2 (10%)
Nitric oxide *n* (%)	8 (40%)
Intra-aortic balloon pump *n* (%)	7 (35%)
HeartMate score	1.64 (1.26-2.27)
INTERMACS profile	
1 *n* (%)	0 (0%)
2 *n* (%)	3 (15%)
2-TCS *n* (%)	3 (15%)
3 *n* (%)	17 (85%)
3-TCS *n* (%)	4 (20%)
Strategy	
Destination therapy *n* (%)	0 (0%)
Bridge-to-candidacy *n* (%)	10 (50%)
Bridge-to-transplant (BTT) *n* (%)	10 (50%)
BTT-likely *n* (%)	4 (20%)
BTT-unlikely *n* (%)	6 (30%)

Abbreviations: BTT, bridge to transplant; CAD, coronary artery disease; CRT, cardiac resynchronization therapy; ICD, implantable cardioverter defibrillator; INTERMACS, Interagency Registry for Mechanically Assisted Circulatory Support; IQR, interquartile range; TCS, temporary circulatory support.

Data as counts (%) or median (IQR).

**Table 2:** Preimplant Echocardiographic, Hemodynamic, and Laboratory Data

Preimplant Data	Total (N = 20)
Echocardiogram	
Left ventricular ejection fraction (%)	25 (21-28)
Left end-diastolic diameter (mm)	69.5 (66-78.5)
Interventricular septum thickness (mm)	8 (7.8-9)
Posterior wall thickness (mm)	8 (8-9)
Severe mitral regurgitation *n* (%)	12 (60%)
Severe tricuspid regurgitation *n* (%)	3 (15%)
Severe right ventricular dysfunction *n* (%)	1 (5%)
Hemodynamic data	
Systolic systemic blood pressure (mm Hg)	110 (102-120)
Mean systemic blood pressure (mm Hg)	80 (76-90)
Diastolic systemic blood pressure (mm Hg)	65 (60-79)
Systolic pulmonary pressure (mm Hg)	50 (35.5-69)
Mean pulmonary pressure (mm Hg)	37 (23.5-40)
Pulmonary vascular resistance (wood)	2 (1.5-3.6)
Capillary pulmonary pressure (mm Hg)	23.5 (19.2-25.7)
Right atrial pressure (mm Hg)	9 (5-17.7)
Cardiac output (liter/min)	4.6 (4.4-4.8)
Cardiac index (liter/min/m^2^)	2.5(2.2-2.7)
Preimplant laboratory data	
Hemoglobin (g/dl)	10.9 (9.6-12)
Hematocrit (%)	33.7 (29.7-35.6)
Urea (mg/dl)	52 (34.5-59)
Creatinine (mg/dl)	1.1 (0.96-1.3)
Sodium (mEq/liter)	135 (130.7-138)
Estimated creatinine clearance (ml/min/1.73 m^2^)	59.5 (54.7-74.7)
AST (U/liter)	23 (13-28.2)
ALT (U/liter)	24 (19-36.5)
Albumin (g/dl)	3.7 (3.2-3.9)
Total bilirubin (mg/dl)	0.7 (0.4-1.2)
INR	1.2 (1.1-1.3)

Abbreviations: ALT, alanine aminotransferase; AST, aspartate aminotransferase; INR, International normalized ratio; IQR, interquartile range.

Data as counts (%) or median (IQR). Laboratory analysis, echocardiogram, and right heart catheterization measures were obtained the day before surgery, and all patients were on inotropes.

### Patient referral

**Figure 1:**
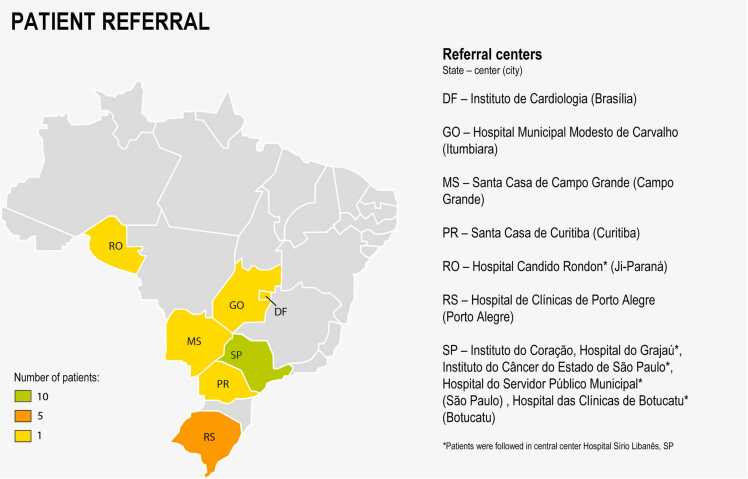
Map of Brazil showing patient referral by state and center.

Patients were referred from 11 centers from 7 states in Brazil (Figure 1). Five of these centers had HF specialists. Additionally, nine other patients were considered for inclusion in the program. However, six had dialysis-dependent renal disease, and three were classified as INTERMACS profile 1, thus leading to their exclusion.

### Surgery data

The devices used in the philanthropy program at our center during the study period were HMII in 9 (45%) patients, HM3 in 9 (45%) patients, and Berlin Heart INCOR in 2 (10%) patients. There were 2 primary operators, and each one performed 10 cases. The median cardiopulmonary bypass time was 110 minutes, and median sternotomy was performed in all patients. Only 1 patient had a concomitant procedure, which was a tricuspid valve repair.

Intraoperative bleeding was common, with 70% of patients requiring at least 1 unit of packed red blood cells (PRBC) transfusion. However, only 3 patients (15%) experienced bleeding necessitating 4 or more units of PRBC. During the first 7 days postoperatively, none of the patients required 4 or more units of PRBC within any 48-hour period. Reoperation due to bleeding was required in 20% of cases. Bleeding events were more common in the initial implants at our center: among the first 10 patients, 8 required blood transfusions and 3 underwent reoperation due to bleeding. In contrast, among the last 10 patients, 6 required blood transfusions and only 1 required reoperation due to bleeding. Reoperation due to cardiac tamponade happened in 2 patients. Additional surgery data are described in Table S1.

### Survival analysis and outcomes

**Figure 2:**
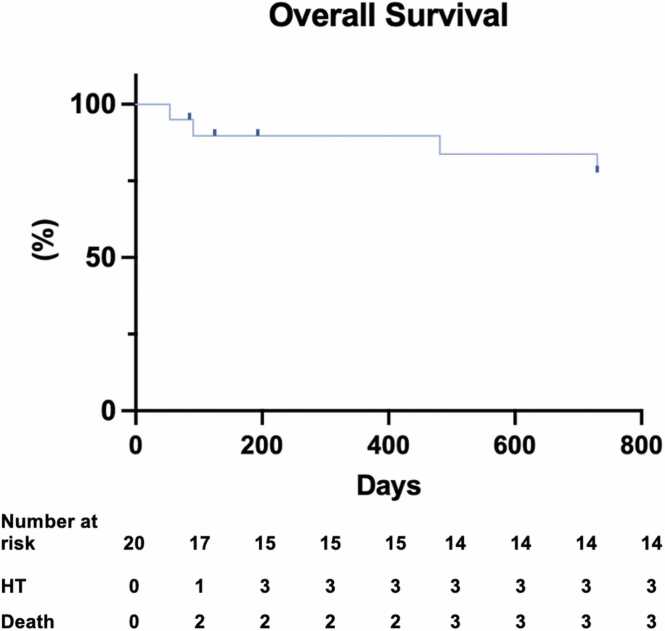
Kaplan-Meier survival analysis. HT, heart transplant.

Outcomes are described in Table 3. The overall survival censored for competing risks at 1 and 2 years were 90% and 84%, respectively (Figure 2). The main cause of death was hemorrhagic stroke in 2 cases and the third patient suffered from septic shock from an LVAD-related infection. Three patients (15%) underwent an HT in the first 2 years after LVAD implantation. Two patients experienced recovery of LVEF, resulting in attempts to discontinue LVAD support. One patient underwent unsuccessful LVAD explant as he died from hemorrhagic shock during the procedure and another patient had the LVAD deactivated using a Left Atrial Appendage Occluder in the Outflow Cannula.^6^

**Table 3:** Outcomes

Outcomes	Total (N = 20)		
Survival censored for competing risks (Kaplan-Meier)			
In 1 year *n* (%)	16 (90%)		
In 2 years *n* (%)	15 (84%)		
Heart transplant *n* (%)	6 (30%)		
LVAD exchange due to pump thrombosis *n* (%)	1 (5%)		
LV function recovery	2 (10%)		
LVAD explant^a^*n* (%)	1 (5%)		
LVAD deactivation *n* (%)	1 (5%)		
Postimplant ICU length of stay (days)	29.5 (24-43.5)		
Postimplant hospital length of stay (days)	27.5 (24-41.2)		
			
Complications	Total	Early (<30 days)	Late (>30 days)
Driveline infection *n* (%)	12 (60%)	2 (10%)	10 (50%)
Sepsis *n* (%)	6 (30%)	4 (20%)	2 (10%)
Nonsurgical bleeding *n* (%)	3 (15%)	0 (0%)	3 (15%)
Ischemic stroke *n* (%)	2 (10%)	0 (0%)	2 (10%)
Hemorrhagic stroke *n* (%)	2 (10%)	0 (0%)	2 (10%)
LVAD thrombosis *n* (%)	2 (10%)	1 (5%)	1 (5%)
Ventricular arrhythmias *n* (%)	6 (30%)	5 (25%)	1 (5%)
Driveline fracture *n* (%)	1 (5%)	0 (0%)	1 (5%)
Right ventricular failure *n* (%)	8 (40%)	8 (40%)	0 (0%)
Right ventricular MCS *n* (%)	1 (5%)	1 (5%)	0 (0%)
Acute renal failure *n* (%)	7 (35%)	7 (35%)	0 (0%)
Hemodialysis *n* (%)	2 (10%)	2 (10%)	0 (0%)
Hepatic insufficiency *n* (%)	1 (5%)	1 (5%)	0 (0%)

Abbreviations: ICU, intensive care unit; IQR, interquartile range; LV, left ventricular; LVAD, left ventricular assist device; MCS, mechanical circulatory support.

Data as counts (%) or median (IQR).

a LVAD explant was unsuccessful since the patient died from hemorrhagic shock during the procedure.

At the end of the data collection period (December 31, 2022), 1 patient was alive with an inactivated HMII, 8 patients were receiving ongoing active LVAD support, including 2 women with Chagas cardiomyopathy, and 6 patients underwent an HT. These Chagas cardiomyopathy patients included a patient implanted via a BTC strategy who had an implantable cardioverter defibrillator and suffered 1 event of sustained early ventricular tachycardia requiring implantable cardioverter defibrillator shock, but these events did not recur after adjustment of antiarrhythmics. She is now with 832 days of ongoing uncomplicated HM3 support at the end of the study follow-up period. The other Chagas cardiomyopathy patient originally received an HMII implant via a BTT-unlikely strategy but later developed a late pump thrombosis requiring exchange to another HMII during the study period, accumulating 1,366 days of total ongoing HMII LVAD support with a late, localized driveline infection of the second LVAD. None of the other 20 patients was diagnosed with Chagas cardiomyopathy.

### Follow-up and complications

After initial postoperative stabilization, 12 out of 20 patients returned to their original centers and were followed according to our protocol described in Materials and methods. Seven patients were followed directly in our center and 1 patient died during the index hospitalization for LVAD implant.

Among LVAD complications (Table 3), driveline infection was the most common (60%). Driveline infection was early in 2 (10%) patients, while 10 (50%) patients developed late driveline infection. Among patients with late driveline infection, 4 were followed in our center, 5 in the regional centers with HF specialists, and 1 patient was followed in a regional center without an HF specialist. This last patient required hospitalization at a regional center for intravenous antibiotics, with a consultation held remotely with the central hospital's HF team. Following discharge, she continued regular outpatient follow-up appointments every 4 months at the central center.

Two patients (10%) presented with pump thrombosis, and both were supported by an HMII. One of them had the device exchanged to another HMII in the central center and the other patient was submitted to HT also in the central center. Three patients (15%) suffered neurological events: 1 presented with a late ischemic stroke followed by hemorrhagic conversion, 1 developed an ischemic stroke, and 1 had an intracerebral hemorrhage.

Three patients (15%) developed nonsurgical bleeding excluding neurological events: one received 1 PRBC transfusion for management-related gastrointestinal bleeding, another patient received 2 PRBC for gastrointestinal bleeding, and the third patient presented epistaxis requiring 1 PRBC transfusion. We only recorded transfusions that occurred at the central implant center, and there are likely additional blood transfusions late after implant that were not captured.

Driveline fracture occurred in 1 patient with an HMII, resulting in pump stoppage. However, at the time of the driveline fracture, the patient had recovered LVEF (55%), and the HF team opted to deactivate the HMII using a left atrial appendage occluder successfully positioned inside the outflow cannula.^6^

Early postoperative right ventricular failure occurred in 40% of patients, but only 1 patient was supported by a right ventricle mechanical support (CentriMag Pump [Abbott Laboratories, Chicago, IL]), and the others were supported with inotropes and/or nitric oxide. No patient used phosphodiesterase-5 inhibitors. Right ventricular failure was defined as failure to wean from inotropic or vasopressor support or inhaled nitric oxide within 14 days following LVAD implantation or having to initiate this support within 30 days of implant for a duration of at least 14 days or need for a right ventricle mechanical support. Late right ventricular failure did not occur during follow-up.

All nonsurgical bleeding events, neurological events and pump thrombosis required hospitalization. Hospitalizations occurred in local centers if the patient was in regional follow-up (12 patients), or in the central center if the patient was followed as outpatient in our center (7 patients). Pump thrombosis hospitalizations requiring surgical intervention (either HT or LVAD exchange) were performed in the central center.

## Discussion

We describe a hub-and-spoke model for LVAD implantation and follow-up in Brazil, a middle-income country that has continental dimensions and financial limitations. Centralizing surgical procedures and decentralizing patient follow-up care enabled patients from different regions in Brazil to benefit from this alternative strategy for advanced HF. It is known centralization of surgical procedures in 1 single center enables improvement in surgical skills and intensive care in the immediate postoperative period, leading to better results. Also, we demonstrate it is possible to follow patients in centers without HF specialists, providing 24 hours a day, 7 days a week, and on-call access to telephone support from an LVAD coordinator, and periodic outpatients visits.

We describe survival rates, censored for competing risks, of 90% and 84% at 1 and 2 years, respectively. These rates reflect a highly selected group of patients, as positive outcomes are crucial for an emerging program in a resource-constrained country. Compared to international data, our patients were younger, with less organ dysfunctions and comorbidities.^2^ Eighty-five percent were INTERMACS profile 3 patients, 15% were INTERMACS profile 2 patients, and there were no INTERMACS profile 1 patients, whereas the INTERMACS annual report of 2022 included 16.5% INTERMACS profile 1 patients and 34.4% INTERMACS profile 2 patients.^1^ Our initial results lay the groundwork for the future inclusion of a larger population with advanced HF in our program, with less stringent inclusion and exclusion criteria.

The limited number of implants is largely due to strict inclusion and exclusion criteria and financial constraints within the philanthropic program, as the Programa de Apoio ao Desenvolvimento Institucional do SUS encompasses various programs within each of its partner philanthropic hospitals.^4^ Thus, the funds derived from tax exemption are not solely allocated to LVAD implants, but also to other programs within our center. Unfortunately, the current landscape in our country regarding the provision of LVAD therapy to patients served by the public health system remains limited. While LVAD implantation is conducted in centers across several states in Brazil, it is predominantly confined to private hospitals and individuals with distinct financial circumstances and specific health insurance coverage due to the high cost associated with this therapy in our country.^3^ There is no official registry for LVADs in Brazil. Our central center implanted an additional 6 LVADs through private care during the timeframe of our study, and based on data provided by Abbott, it is estimated that approximately 72 LVAD implants were performed in Brazil during this period, mostly HMII and HM3, but also Berlin Heart INCOR and Medtronic/HeartWare HVAD (HVAD, Medtronic Inc., Minneapolis, MN).

Data regarding LVAD implantation in middle-income countries are limited. Case series from single institutions have been published by 1 center in Chile and another in Serbia.^7^, ^8^ Both groups selected less critical patients, though in different ways. In the Chilean case series, 5 out of 9 patients were classified as INTERMACS profile 4,^8^ while in the Serbian case series, 64% of the patients were classified as INTERMACS profiles 4 or 5.^7^ Additionally, the challenge of accessing LVADs in middle-income countries extends beyond cost issues. Areas distant from major centers lack surgical facilities, necessitating a hub-and-spoke strategy, as described in island states.^9^ This approach emphasizes the need to centralize complex care in larger centers while decentralizing other aspects of care to regional centers.

Two of our patients had Chagas cardiomyopathy, which is a common etiology for HF in Latin America and a challenge for developed countries because of migration.^10^ The literature is scarce regarding LVAD and Chagas disease. The first case of a patient with Chagas cardiomyopathy undergoing LVAD implantation as a BTT was described in Brazil in 1994,^11^ and, since then, there a few case reports using HMII as BTC^12^ or BTT.^13^, ^14^ This etiology of HF is usually associated with biventricular dysfunction, often with ventricular arrhythmias, making it uninviting for LVAD implantation. However, our 2 Chagas cardiomyopathy patients did not have severe right ventricular dysfunction nor limiting ventricular arrhythmias before LVAD implantation and have remained free of those specific complication for their sustained period of LVAD support to date.

Most of our surgical complications were related to the learning curve. Bleeding requiring transfusion was frequent in our study, but most of the bleeding cases occurred in the first implants. Cardiac tamponade requiring drainage also occurred in 2 of the first 5 patients, and after those, there were no other cases. Those initial results certainly impact in a longer intensive care unit stay compared to literature.^15^ Also, we had 40% rate of right ventricular failure, and these patients used inotropes for more than 14 days, which might have contributed to a longer period in intensive care unit.^16^, ^17^

LVAD-related infection was very frequent in our study, occurring in 60% of all patients, and it happened independently of the follow-up being performed in our center or in the regional center. This percentage is much higher than the reported 17.5% in the INTERMACS annual report.^1^ To mitigate this problem, we began to use adhesive tube attachment devices to secure the driveline, providing lateral stabilization. We believe the main reasons for the high number of driveline infections are related to the learning curve of our center, the need for additional training of central and regional teams, patients and caregivers, and the relatively high cost of appropriate cleaning materials led to limited access for patients.

### Clinical implications

Our results encourage the expansion of hub-and-spoke models for LVAD implantation among countries with similar socioeconomic conditions to Brazil. Even with large geographic distances between referring and implanting centers, successful follow-up was achieved. We experienced local challenges especially regarding driveline infection, highlighting the need for promoting training of health care professionals and patients and caregivers to improve patient care. It is our desire that our current report describing LVAD implantation via a hub-and-spoke program in a middle-income country spanning a large geographical territory, and funded by a philanthropic and tax-incentive scheme, will help inform future efforts to minimize the current inequitable access to LVAD implantation.

### Limitations

This study has limitations. Data were collected retrospectively, leading to limitations intrinsic to this modality of study. The nonsurgical bleeding events are likely undercaptured. The number of patients is low considering the follow-up period. Finally, although the follow-up was performed in different centers, all the implants were performed at a single center, and therefore may not be representative of outcomes in other centers in Brazil and Latin America.

## Conclusion

This study describes a successful LVAD implantation program in a spoke-and-hub model in Brazil for a carefully selected cohort of patients, and funded via a philanthropic effort in which costs were defrayed by a hospital tax exemption program. This system exists in parallel with LVADs implanted at this and other centers receiving reimbursement via private insurance or cash payment. Centralization of LVAD implants and decentralization of postimplant patient care in regional centers is feasible and safe in a geographically expansive area and enables optimal allocation of resources, which is pivotal for lower- and middle-income countries. Further efforts in middle-income countries to decrease cost, improve access, minimize inequities, and improve economic viability of this life-saving technology are needed.

## Author contributions

Deborah de Sá Pereira Belfort: writing – original draft, investigation, data curation, formal analysis. Bruno Biselli: investigation, data curation, writing – review and editing, validation. Mônica Samuel Avila: investigation, data curation, writing – review and editing, validation. Renata Lopes Hames: investigation, data curation. Stephanie Itala Rizk: investigation, data curation. Fabrício Canova Calil: investigation, data curation. Bruna Carneiro Oliveira: investigation, data curation. Filomena Regina Barbosa Gomes Galas: data curation. Ludhmila Abrahão Hajjar: data curation. Nadine Oliveira Clausell: investigation, supervision. Livia Adams Goldraich: investigation, data curation, writing – review and editing. Ramez Anbar: data curation. Edimar Alcides Bocchi: writing – review and editing, validation. Tadeu Thomé: resources, project administration. Roberto Kalil Filho: supervision, project administration. Paulo Manuel Pêgo-Fernandes: supervision, project administration. Fabio Biscegli Jatene: supervision, project administration. Silvia Moreira Ayub-Ferreira: conceptualization, methodology, writing – review and editing, supervision, project administration.

## Disclosure statement

The authors declare the following financial interests/personal relationships which may be considered as potential competing interests: Silvia Moreira Ayub-Ferreira and Bruno Biselli report administrative support was provided by Abbott. Silvia Moreira Ayub-Ferreira and Bruno Biselli report a relationship with Abbott that includes nonfinancial support. The other authors declare that they have no known competing financial interests or personal relationships that could have appeared to influence the work reported in this paper.

We acknowledge Abbott for their invaluable support in implementing our long-term device program. Abbott provided in-kind program development advice and in-kind support, including standard-of-care training for selected site health care staff and travel support including travel to the Abbott Users Meeting for selected personnel. Abbott did not provide any direct financial support and had no role in the writing and development of this academic manuscript.

Financial support was provided by the 10.13039/501100006506Brazilian Ministry of Health, through Support Program for Institutional Development of the Unified Health System (Programa de Apoio ao Desenvolvimento Institucional do Sistema Único de Saúde - Proadi-SUS).

**Figure:**
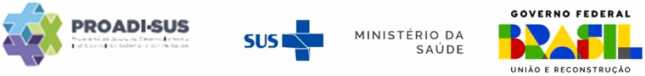

.

## Data Availability

The data underlying this article are available in the article and its online Supplementary Material. Additional data that support the findings of this study are available upon request.

## References

[1] Yuzefpolskaya M., Schroeder S.E., Houston B.A. (2023). The Society of Thoracic Surgeons Intermacs 2022 Annual Report: focus on the 2018 heart transplant allocation system. Ann Thorac Surg.

[2] McNamara N., Narroway H., Williams M. (2021). Contemporary outcomes of continuous-flow left ventricular assist devices-a systematic review. Ann Cardiothorac Surg.

[3] Ayub-Ferreira S.M., Biselli B. (2022). Long-term ventricular assist devices: where are we in Brazil?.

[4] Santos J.A., Palhares L., Mendes A. (2023). Proadi-SUS: analysis of financial resources in the three-year periods 2009-2011, 2012-2014 and 2015-2017. Rev Saude Publ.

[5] Kormos R.L., Antonides C.F.J., Goldstein D.J. (2020). Updated definitions of adverse events for trials and registries of mechanical circulatory support: a consensus statement of the mechanical circulatory support academic research consortium. J Heart Lung Transplant.

[6] Pereira A.B., Belfort D.S.P., Biselli B. (2023). A novel percutaneous technique for left ventricular assist device deactivation using a left atrial appendage occluder in the outflow cannula. ASAIO J.

[7] Nestorovic E., Schmitto J.D., Kushwaha S.S. (2018). Successful establishment of a left ventricular assist device program in an emerging country: one year experience. J Thorac Dis.

[8] Pedemonte O., Vera A., Merello L. (2018). Left ventricular assist device (LVAD) program in Chile: first successful experience in South America. J Thorac Dis.

[9] Vinck E.E., Vervoort D., Tiwari K.K. (2022). Destination left ventricular assist devices in island states: asking too much or the inevitable solution. Cardiothorac Surg.

[10] Bocchi E. (2018). Chagas disease cardiomyopathy treatment remains a challenge. Lancet.

[11] Bocchi E.A., Vieira M.L., Fiorelli A. (1994). Hemodynamic and neurohormonal profile during assisted circulation with heterotopic artificial ventricle followed by heart transplantation. Arq Bras Cardiol.

[12] Atik F.A., Cunha C.R., Chaves R.B., Ulhoa M.B., Barzilai V.S. (2018). Left ventricular assist device as a bridge to candidacy in end-stage chagas cardiomyopathy. Arq Bras Cardiol.

[13] Kransdorf E.P., Czer L.S., Luthringer D.J. (2013). Heart transplantation for chagas cardiomyopathy in the United States. Am J Transplant.

[14] Ruzza A., Czer L.S., De Robertis M. (2016). Total artificial heart as bridge to heart transplantation in chagas cardiomyopathy: case report. Transplant Proc.

[15] Goldstein D.J., Meyns B., Xie R. (2019). Third annual report from the ISHLT Mechanically Assisted Circulatory Support Registry: a comparison of centrifugal and axial continuous-flow left ventricular assist devices. J Heart Lung Transplant.

[16] Akin S., Soliman O., de By T.M.M.H. (2020). Causes and predictors of early mortality in patients treated with left ventricular assist device implantation in the European Registry of Mechanical Circulatory Support (EUROMACS). Intensive Care Med.

[17] Cotts W.G., McGee E.C., Myers S.L. (2014). Predictors of hospital length of stay after implantation of a left ventricular assist device: an analysis of the INTERMACS registry. J Heart Lung Transplant.

