# Corrigendum to “Development of a hub-and-spoke durable left ventricular assist device program in Brazil, a middle-income country” [JHLT Open, 6 (2024) 100151]

**DOI:** 10.1016/j.jhlto.2024.100193

**Published:** 2024-12-21

**Authors:** Deborah de Sá Pereira Belfort, Bruno Biselli, Mônica Samuel Avila, Renata Lopes Hames, Stephanie Itala Rizk, Fabrício Canova Calil, Bruna Carneiro Oliveira, Elaine Marques Hojaij, Filomena Regina Barbosa Gomes Galas, Ludhmila Abrahão Hajjar, Nadine Oliveira Clausell, Livia Adams Goldraich, Ramez Anbar, Edimar Alcides Bocchi, Tadeu Thomé, Roberto Kalil Filho, Paulo Manuel Pêgo-Fernandes, Fabio Biscegli Jatene, Silvia Moreira Ayub-Ferreira

**Affiliations:** a Hospital Sirio-Libanes, São Paulo, SP, Brazil; b Heart Institute (InCor), Instituto do Coração, Hospital das Clinicas, HCFMUSP, Universidade de São Paulo, São Paulo, SP, Brazil; c Hospital de Clinicas de Porto Alegre, Universidade Federal do Rio Grande do Sul, Porto Alegre, RS, Brazil

The authors regret to inform that an important contributor to the work was omitted from the original publication. We sincerely apologize for not including Elaine Marques Hojaij, a key member of the multidisciplinary team, who played a fundamental role in the development of the program. Elaine Marques Hojaij, a psychologist who contributed to all the cases, was essential in the areas of data curation, investigation, and visualization. We deeply regret this oversight and wish to give full credit to her invaluable input in the project as one of the authors.

The authors would like to apologize for any inconvenience caused.

