# Supplementary material for: Development of a hub-and-spoke durable left ventricular assist device program in Brazil, a middle-income country
Source: JHLT Open. 2024 Aug 23;6:100151. doi: 10.1016/j.jhlto.2024.100151 (PMC11935476; doi:10.1016/j.jhlto.2024.100151)
Supplement: Supplementary file 1 — Supplementary material [file mmc1.docx]

| Table S1. Surgery data |  |
| --- | --- |
| LVAD implant data | Total (N=20) |
| Extracorporeal circulation time (minutes) | 110 (95-118.7) |
| Bleeding requiring blood transfusion during surgery n(%) | 14 (70%) |
| Bleeding requiring reoperation n(%) | 4 (20%) |
| Tamponade n(%) | 2 (10%) |
| Type of LVAD |  |
| HeartMate 3™ n(%) | 9 (45%) |
| HeartMate II™ n(%) | 9 (45%) |
| Berlin Heart INCOR® n(%) | 2 (10%) |
| Data as counts (%) or median (IQR). ICU: intensive care unit; LVAD: left ventricular assist device. | |
